# Force-controlled robotic ultrasound elastography enhances diagnostic consistency for thyroid nodules

**DOI:** 10.1186/s13244-025-02181-5

**Published:** 2026-04-16

**Authors:** Tianhui Yan, Xu Le, Li Xie, Xiangyu Qiu, Delei Cheng, Chong Pei, Lei Hu

**Affiliations:** 1 Department of Ultrasound, The First Affiliated Hospital of USTC, Division of Life Sciences and Medicine, University of Science and Technology of China, Hefei, China; 2 Department of Interventional Radiology, The First Affiliated Hospital of USTC, Division of Life Sciences and Medicine, University of Science and Technology of China, Hefei, China; 3 Department of Respiratory and Critical Care Medicine, The First People’s Hospital of Hefei City, The Third Affiliated Hospital of Anhui Medical University, Hefei, China

**Keywords:** Force-controlled robotic arm, Thyroid nodule, Ultrasound elastography, Strain elastography, Shear wave elastography

## Abstract

**Objective:**

To assess whether a force-controlled robotic arm can improve image quality, consistency, and diagnostic accuracy of strain elastography (SE) and shear wave elastography (SWE) in differentiating benign from malignant thyroid nodules.

**Materials and methods:**

In this prospective study, 131 thyroid nodules were examined by a junior physician, a senior physician, and a robotic arm with a PID-based force feedback system. Image quality was evaluated using the structural similarity index (SSIM), peak signal-to-noise ratio (PSNR), mean squared error (MSE) and mean opinion score (MOS). Consistency was assessed with the Dice similarity coefficient (DSC) and the intraclass correlation coefficient (ICC) for SWE-derived Emax. Diagnostic performance was analyzed via ROC curves and AUC comparisons.

**Results:**

The robotic arm achieved higher image quality (SSIM up to 0.94, PSNR 42.25 dB, MSE 3.06) and MOS scores (SE: 4.1 ± 0.3; SWE: 4.3 ± 0.2) than both human operators (all *p* < 0.001). It also showed better consistency (DSC up to 0.90; ICC up to 0.94) and diagnostic accuracy (AUC 0.90 for SE, 0.95 for SWE; *p* < 0.05).

**Conclusion:**

The force-controlled robotic arm provides standardized, reproducible thyroid elastography with superior quality, consistency, and accuracy compared with manual scanning.

**Critical relevance statement:**

This study explores robotic-assisted elastography to improve consistency in thyroid nodule assessment.

**Key Points:**

- The force-controlled robotic arm enhanced ultrasound elastography by generating clearer, more standardized images compared with manual acquisition.
- The force-controlled robotic arm scanning achieved superior intra-operator reproducibility.
- Diagnostic performance in differentiating benign from malignant thyroid nodules was significantly improved with robotic elastography, exceeding that of both junior and senior radiologists.

**Graphical Abstract:**

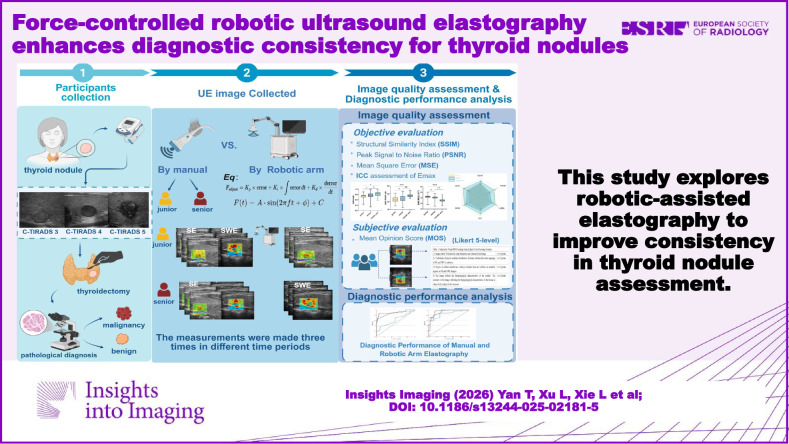

## Introduction

Ultrasound elastography is a noninvasive imaging technique that quantitatively evaluates tissue stiffness and plays an increasingly important role in characterizing focal lesions, particularly in the liver, breast, and thyroid [1–3]. By assessing differences in tissue elasticity, elastography effectively differentiates benign from malignant nodules, thereby enhancing diagnostic confidence [4].

Clinically, two primary elastography methods are employed: strain elastography (SE), which measures relative tissue deformation under external compression, and shear wave elastography (SWE), which quantitatively assesses tissue stiffness based on shear wave propagation velocity. Both SE and SWE critically depend on stable, consistent force application during scanning. However, manual force variability among radiologists reduces reproducibility and accuracy: inconsistent compression in SE causes nonstandardized deformation, while unstable transducer-tissue contact in SWE compromises shear wave quality [5–7].

Although conventional elastography has demonstrated diagnostic utility for thyroid nodules, radiologist-dependent variability remains a significant limitation. Even experienced radiologists apply inconsistent pressure across examinations, resulting in decreased measurement reliability and diagnostic performance. Consequently, the integration of SE and SWE into established thyroid risk stratification systems remains limited [8–10]. Robotic telesonography systems offer potential for stable force application in ultrasound elastography; however, most current platforms lack real-time force control [11–13]. To address this, we developed a robotic arm with real-time force feedback to ensure standardized and reproducible tissue compression during elastography [14, 15].

The purpose of this study was to evaluate the effectiveness of this force-controlled robotic arm in thyroid elastography. Specifically, we aimed to assess its performance in terms of image quality, intra-group consistency, and diagnostic accuracy for differentiating benign from malignant thyroid nodules [16]. We also sought to determine its potential to support standardized and reproducible ultrasound workflows, ultimately enabling more reliable and objective clinical decision-making.

## Materials and methods

### Study design and participants

This prospective study enrolled patients with thyroid nodules who underwent ultrasound evaluation in the Ultrasound Diagnostic Clinic of our hospital between August 2024 and December 2024. Each thyroid nodule was evaluated by SE and SWE, performed independently by junior physicians (with 5 years of elastography experience), senior physicians (with 10 years of elastography experience), and a force-controlled robotic arm.

**Fig. 1:**
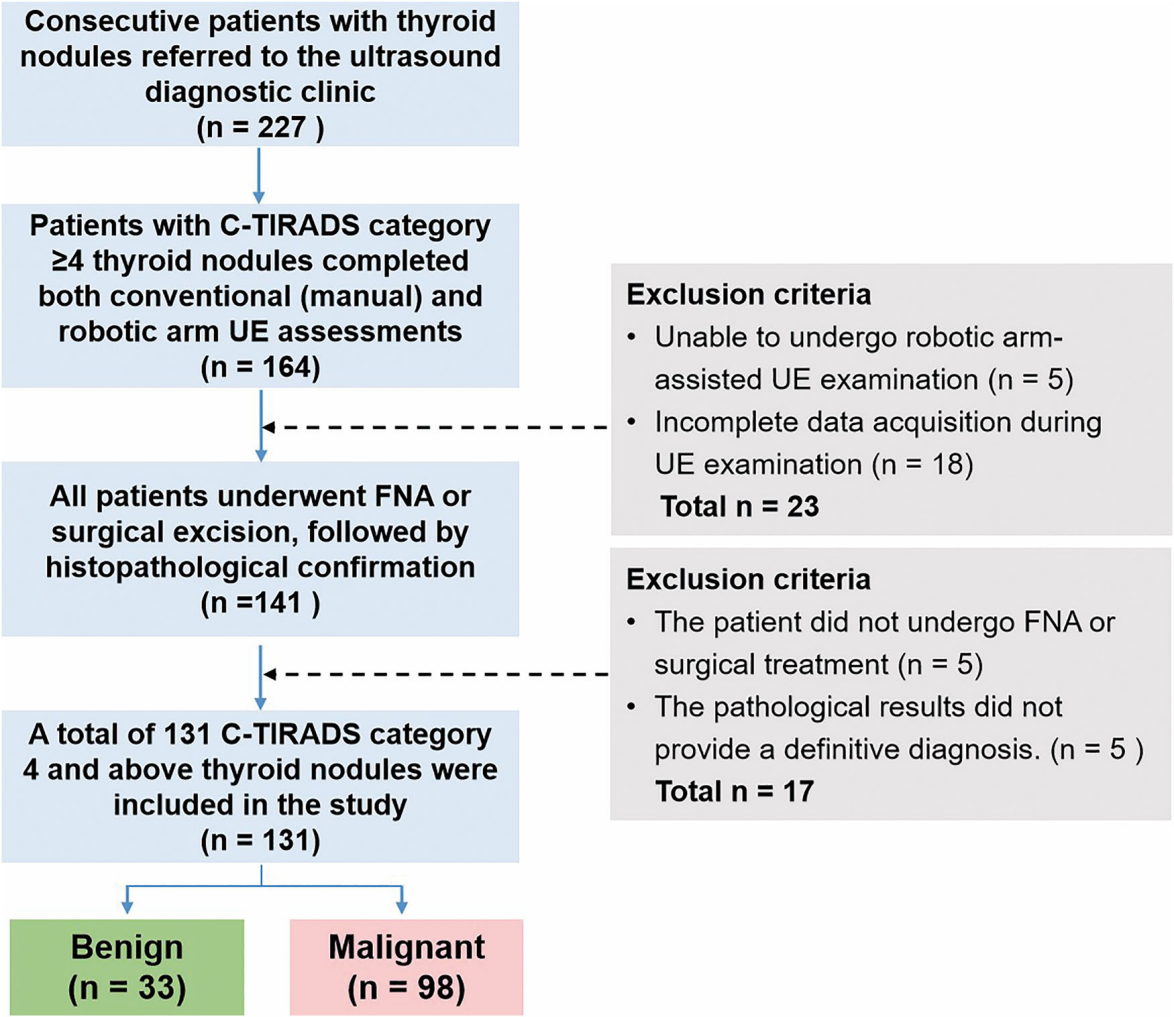
Flowchart of patient selection

This study was approved by the Ethics Committee of the First Affiliated Hospital of the University of Science and Technology of China (approval No. 2023KY333). All patients provided written informed consent prior to participation. All nodules had definitive pathological diagnoses. Inclusion Criteria: (1) Patients with thyroid nodules who underwent both conventional ultrasound and ultrasound elastography; (2) Nodules with definitive cytological diagnoses obtained by fine-needle aspiration (FNA) or histopathological diagnoses confirmed by surgery, along with complete clinical data. Exclusion Criteria: (1) Patients unable to tolerate ultrasound examination or robotic-arm operation; (2) Nodules with a diameter smaller than 5 mm, precluding effective elastography evaluation. A flowchart of patient recruitment is presented in Fig. 1.

### Force-controlled robotic arm and ultrasound imaging platform

The force-controlled robotic arm used in this study was a custom-designed 7-degree-of-freedom (7-DOF) system developed specifically for ultrasound elastography applications. Integrated with high-precision force sensors and a real-time control algorithm, the system enabled stable, precise, and repeatable pressure application under physician supervision. Both the probe trajectory and applied compression force were tightly regulated to ensure consistent tissue contact throughout the examination, thereby improving the standardization and reproducibility of elastographic image acquisition.

Ultrasound examinations were performed using the Mindray Resona R9s system, equipped with a high-frequency linear array transducer (5–15 MHz) and a dedicated thyroid imaging module. The system provided high-resolution B-mode imaging for detailed visualization of thyroid anatomy and small lesions. In elastography mode, it supported both SE and SWE, enabling real-time acquisition of elastographic data and quantitative assessment of strain ratios and Young’s modulus. All equipment was calibrated before the study to ensure consistency and measurement accuracy across all examinations.

### Robotic arm force control settings

In this study, a force-controlled robotic arm was used for ultrasound elastography, which improved the stability and reproducibility of imaging by precisely controlling the force applied by the probe (Supplementary Fig. S1). The system consists of a seven-degree-of-freedom robotic arm, a six-dimensional force/torque sensor (6DF-TS) and a PID control system, where the PID control system regulates the force application based on feedback to ensure consistency (Supplementary Figs. S2, S3). The robotic arm monitors the force applied by the probe in real time through a six-dimensional force/torque sensor and uses the PID control system to adjust the output of the force to make it conform to the target value. PID control as formula (1) :

1 $${F}_{{{\rm{adjust}}}}={K}_{P}\times error+{K}_{i}\times {\int }errordt+{K}_{d}\times \frac{derror}{dt}$$

In particular, the PID controller adjusts the output of the force to ensure that the applied force is stable due to the deviation between the target force and the real-time measured force. The system can optimize the force consistency of ultrasound elastography, improve image quality and measurement reliability, and provide a standardized force application scheme for ultrasound robots in clinical applications, which helps to improve the accuracy and repeatability of ultrasound diagnosis.

Two force control strategies were designed for different imaging modes. In SE mode, a sine wave force is applied to the robotic arm, and its target force is expressed as a formula (2):

2 $$F(t)=A\cdot \,\sin (2\pi ft+\phi )+C$$

In SWE mode, the target force is constant, and the PID controller adjusts according to the error between the applied force and the target constant force to ensure stable propagation of the shear wave, reduce the measurement error, improve the repeatability of the SWE image, and apply the force as formula (3):

3 $$F(t)={K}_{p}\cdot e(t)+{K}_{i}\cdot {\int }e(t)dt+{K}_{d}\cdot \frac{de(t)}{dt}$$

### Ultrasonic elastography image acquisition procedures

Standard reference acquisition: A senior radiologist with over 15 years of experience in ultrasound elastography performed multiple SE and SWE scans for each thyroid nodule. The highest-quality images—selected based on clarity, stability, and diagnostic interpretability—were reviewed independently by a second senior radiologist. Only images jointly confirmed as excellent were included as standard references for subsequent comparative analyses.

Manual acquisition by physicians: For manual acquisition, the radiologist first activated SE mode and applied rhythmic manual compression to obtain stable strain images. Subsequently, in SWE mode, the probe was held steady to acquire shear wave images. If image quality was suboptimal, the scan was repeated to ensure diagnostic adequacy.

Force-controlled robotic arm acquisition: In the force-controlled robotic arm group, the force-controlled system automatically identified and locked onto the target nodule. Using a predefined scanning trajectory and real-time force feedback, the robotic arm performed SE and SWE acquisitions autonomously. Images were auto-frozen upon meeting stabilization criteria to ensure consistent and high-quality outputs (Supplementary Videos 1–4).

**Fig. 2:**
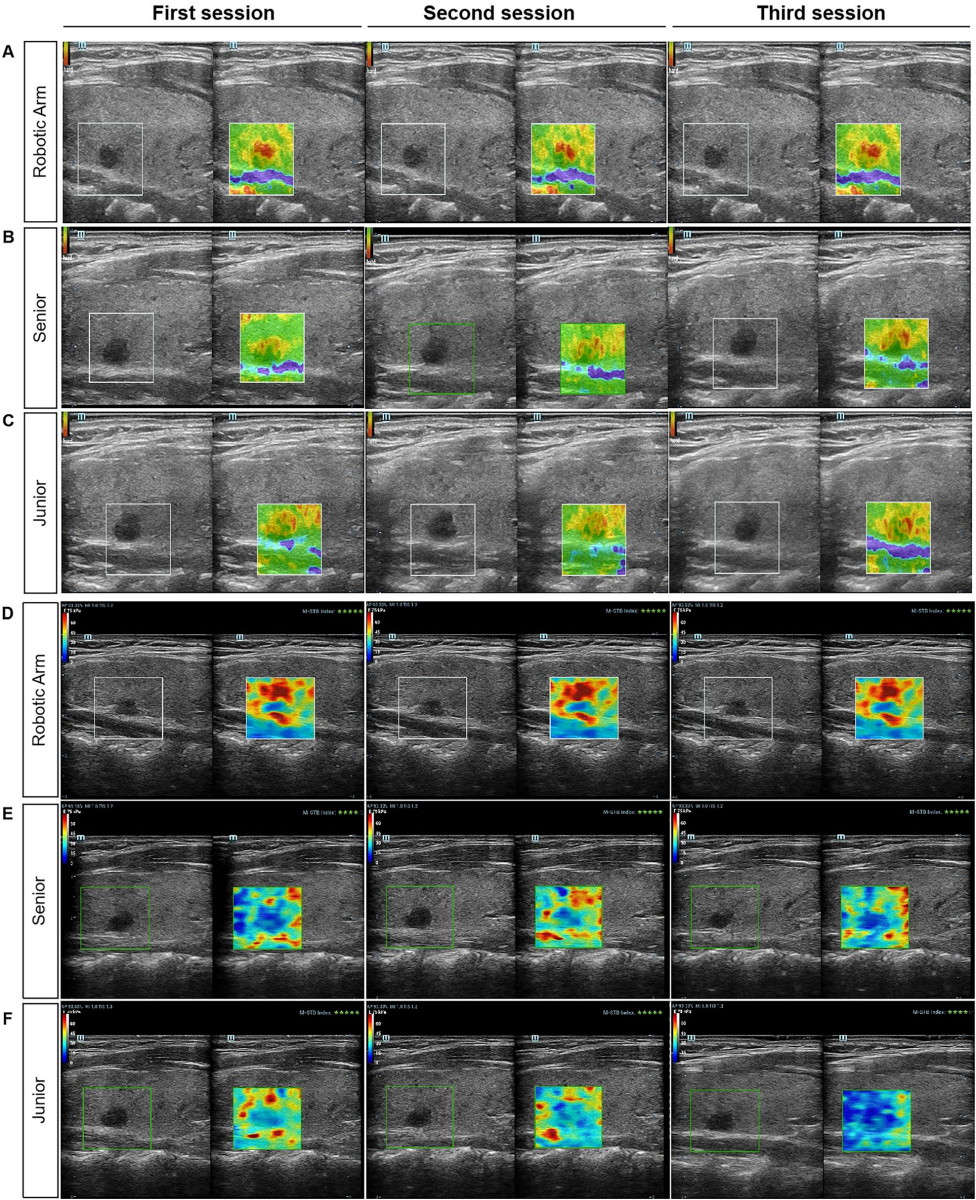
Repeated strain elastography (SE) and shear wave elastography (SWE) images of the same thyroid nodule acquired by three operator groups. **A**–**C** SE images acquired by the junior physician (**A**), senior physician (**B**), and robotic arm (**C**); **D**–**F** SWE images acquired by the junior physician (**D**), senior physician (**E**), and robotic arm (**F**). Each row shows three repeated acquisitions of the same nodule

Examination protocol: Each thyroid nodule underwent three full SE and SWE imaging sessions—performed independently by a junior physician, a senior physician, and the robotic arm. All images and quantitative data were archived for subsequent analysis. Patient safety was continuously monitored, and examinations were immediately terminated if any discomfort occurred (Fig. 2). Operator-specific workflows and operation times (robotic-T, senior-T, junior-T) are detailed in Appendix E1.

### Image quality and reproducibility evaluation

To evaluate image quality, SE and SWE images obtained by junior radiologists, senior radiologists, and the robotic arm were compared against standard reference images using three objective metrics: structural similarity index (SSIM), peak signal-to-noise ratio (PSNR), and mean squared error (MSE). Definitions and computation of SSIM, PSNR, and MSE are provided in Appendix E2. Subjective image quality was also assessed by an experienced radiologist, blinded to operator identity, using a 5-point Likert scale for mean opinion score (MOS), based on clarity, contrast, and anatomical boundary delineation. Detailed MOS scoring criteria are provided in Supplementary Table S1.

For reproducibility analysis, the Dice similarity coefficient (DSC) was used to assess spatial overlap of repeated image segmentations, while the intraclass correlation coefficient (ICC) quantified the consistency of maximum elasticity (Emax) measurements across repeated SWE acquisitions. Diagnostic performance in differentiating benign from malignant thyroid nodules was analyzed using receiver operating characteristic (ROC) curves, and area under the curve (AUC) values were calculated for each group. Finally, all quantitative metrics (SSIM, PSNR, 1–MSE, MOS, DSC, and ICC) were normalized and averaged to generate a composite performance profile, which was visualized using radar plots to facilitate multidimensional comparison among the three operator groups. Definitions of all evaluation metrics are summarized in the Supplementary Information.

### Statistical analysis

All data analysis was done in SPSS 26.0 or Python. The Shapiro–Wilk test is used to assess the normality of the data: normally distributed data are represented as mean ± standard deviations, and non-normally distributed data are expressed as median (interquartile range). All statistical tests were two-sided, with *p* < 0.05 indicating that the difference was statistically significant. Taking the standard image as a reference, SSIM, PSNR and MSE were used to objectively evaluate the image quality obtained by each radiologist, and MOS was used for artificial blind scoring. The difference in scores between groups was based on the normality results, and the one-way ANOVA or Kruskal–Wallis test was used. Pairwise comparisons between groups were supplemented by the Tukey HSD test or the Dunn test in the nonparametric method. The spatial structure consistency of the images was evaluated by the DSC. The consistency of Emax in the SWE images acquired by the radiologist at different time points was calculated by the ICC. Based on the postoperative pathological results, the ROC curves of low-seniority, high-seniority physicians and force-controlled robotic arm for the diagnosis of benign and malignant thyroid nodules in SE and SWE modes were plotted, and the AUC was calculated to evaluate its ability to distinguish between them. The difference in AUC between the methods was compared by the DeLong test.

## Results

### General clinical characteristics of included thyroid nodules

A total of 131 consecutive patients with thyroid nodules were prospectively enrolled and stratified by C-TIRADS category and pathological diagnosis, as summarized in Table 1. There were no significant differences in gender distribution among the groups (male proportion: 40.0%, 23.6%, and 17.4%; *p* = 0.23, 14, and 0.46, respectively), nor in mean patient age (47.9 ± 5.6, 46.8 ± 6.3, and 47.8 ± 6.8 years; all *p* > 0.05). Histopathological analysis confirmed papillary thyroid carcinoma (PTC) as the predominant malignant subtype. As the C-TIRADS category increased, the maximum diameter (MD) of nodules showed a significant decreasing trend (from 15.2 ± 4.2 mm in C-TIRADS 3 to 9.8 ± 2.4 mm in C-TIRADS 5; *p* < 0.01), while the proportion of malignant nodules increased markedly (from 20.0% in C-TIRADS 3 to 95.7% in C-TIRADS 5; *p* < 0.01).

**Table 1:** General clinical characteristics of thyroid nodules in the group

Parameters	C- TIRADS 3 (*n* = 30)	C-TIRADS 4 A/B/C (*n* = 55)	C-TIRADS 5 (*n* = 46)	*P1*	*P2*	*P3*
Male/female	12/18	13/42	8/38	0.23	0.14	0.46
Age (years)	47.9 ± 5.6	46.8 ± 6.3	47.8 ± 6.8	0.68	0.56	0.21
MD (mm)	15.2 ± 4.2	11.5 ± 2.1	9.8 ± 2.4	< 0.01	< 0.05	< 0.01
Malignant proportion (%)	6/30	48/55	44/46	< 0.01	< 0.01	< 0.05
Pathological pattern	PTC (*n* = 6)	PTC (*n* = 46) FTC (*n* = 2)	PTC (*n* = 38) FTC (*n* = 6) MTC (*n* = 2)	-		

*P1*: C-TIRADS 3 vs 4; *P2*: C-TIRADS 3 vs 5; *P3*: C-TIRADS 4 vs 5

*MD* maximum diameter of the thyroid nodule, *PTC* papillary thyroid carcinoma, *FTC* follicular thyroid carcinoma, *MTC* medullary thyroid carcinoma

### Image quality evaluation of SE and SWE across radiologist groups

**Fig. 3:**
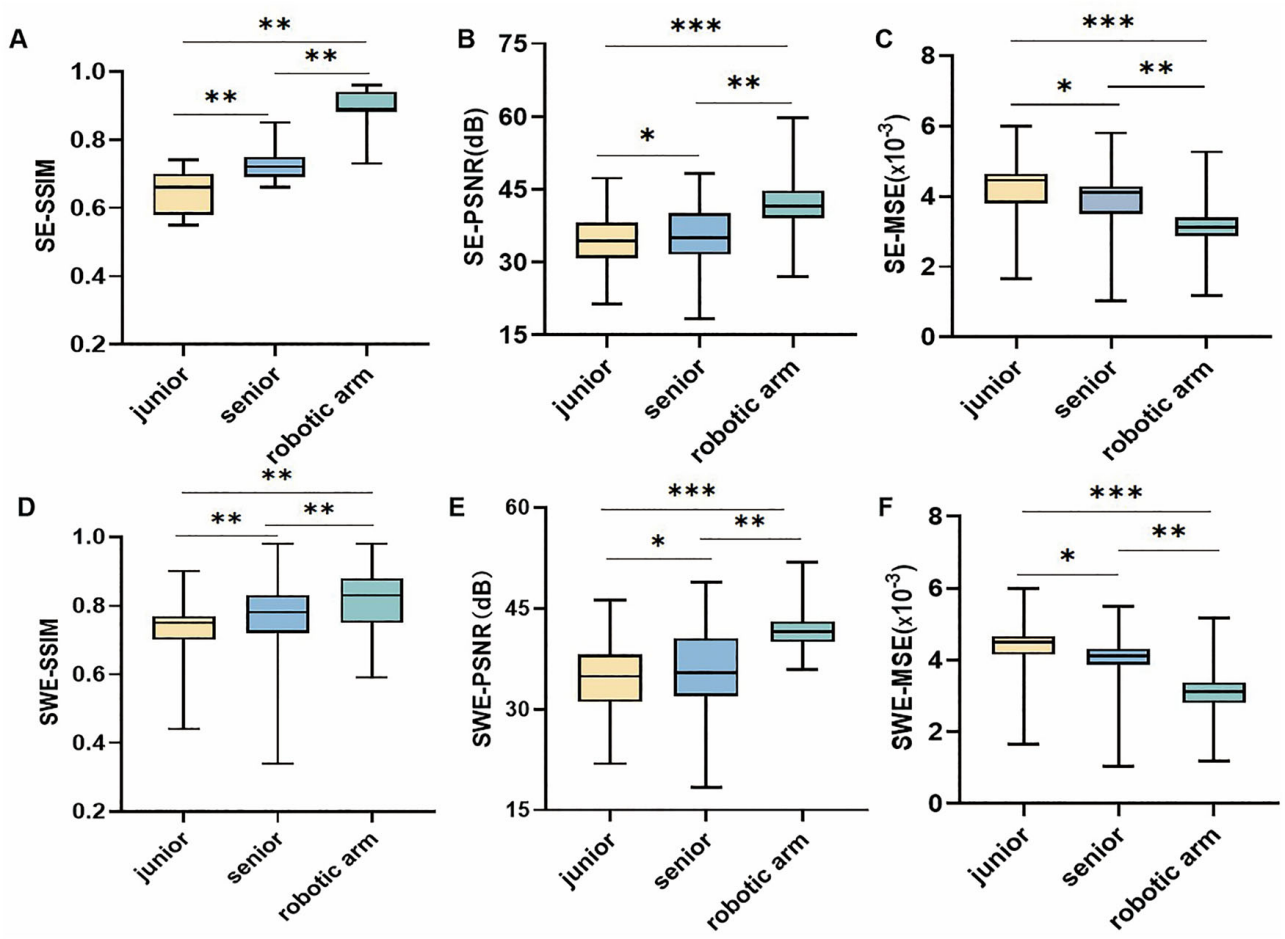
Comparison of image quality metrics among junior, senior, and robotic arm groups. **A**–**C** SWE image quality comparison in terms of SSIM (**A**), PSNR (**B**), and MSE (**C**); **D**–**F** SE image quality comparison in terms of SSIM (**D**), PSNR (**E**), and MSE (**F**). Box plots show the distribution of values for each metric in the three groups. Statistical comparisons were performed using one-way ANOVA followed by Tukey’s HSD test. * *p* < 0.05, ** *p* < 0.01, *** *p* < 0.001

In SE images (Fig. 3A–C), the mean SSIM values were 0.64 (junior), 0.72 (senior), and 0.89 (robotic arm), with significant differences among groups (F = 684.29, *p* < 0.001). The mean PSNR values were 34.36 dB, 35.73 dB, and 42.25 dB, respectively (F = 69.45, *p* < 0.001). The mean MSE values were 4.21 × 10⁻³, 3.84 × 10⁻³, and 3.08 × 10⁻³, respectively (*F* = 60.39, *p* < 0.001). Multiple comparisons showed significant differences between all groups in SSIM, PSNR, and MSE (all *p* < 0.001). In SWE images (Fig. 3D–F), the mean SSIM values were 0.72 (junior), 0.76 (senior), and 0.81 (robotic arm), with significant group differences (*F* = 39.65, *p* = 0.004). PSNR values were 34.75 dB, 36.02 dB, and 41.63 dB, respectively (*F* = 74.58, *p* < 0.001), and the robotic arm group was significantly higher than both manual groups (*p* < 0.001 and *p* < 0.01). MSE values were 4.33 × 10⁻³, 3.92 × 10⁻³, and 3.06 × 10⁻³, respectively (*F* = 94.46, *p* < 0.001), with significant differences between all groups (*p* < 0.001).

### Subjective evaluation of SE and SWE images of the three groups of radiologists

**Fig. 4:**
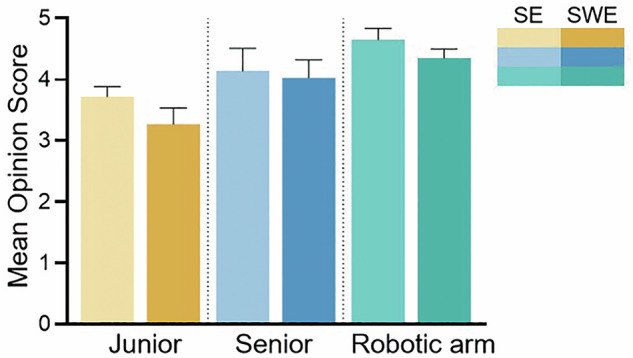
Comparison of the mean diagnostic confidence scores (Mean Opinion Score, MOS) for SE and SWE images acquired by junior physicians, senior physicians, and the robotic arm. The robotic arm group showed the highest average MOS in both SE and SWE modes

The results of MOS showed that the manipulator group was significantly better than the manual radiologist in both SE and SWE modes (Fig. 4). In the SE image score, the robotic arm group also obtained the highest score (4.1 ± 0.3), which was significantly higher than that of the senior physician group (3.7 ± 0.4) and the junior physician group (3.2 ± 0.5), and the difference between the groups was also significant (*F* = 45.62, *p* < 0.001), and the multiple comparisons showed that the difference between the robotic arm group and the manual group was significant (*p* < 0.01). The MOS scores of SWE images were 4.3 ± 0.2 in the robotic arm group, 3.9 ± 0.3 in the senior physician group, and 3.5 ± 0.4 in the junior physician group, with significant differences between the groups (*F* = 52.18, *p* < 0.001), and the differences between the robotic arm group and the other two groups were statistically significant (all *p* < 0.01).

### Intra-group consistency of SE and SWE images across radiologist groups

**Fig. 5:**
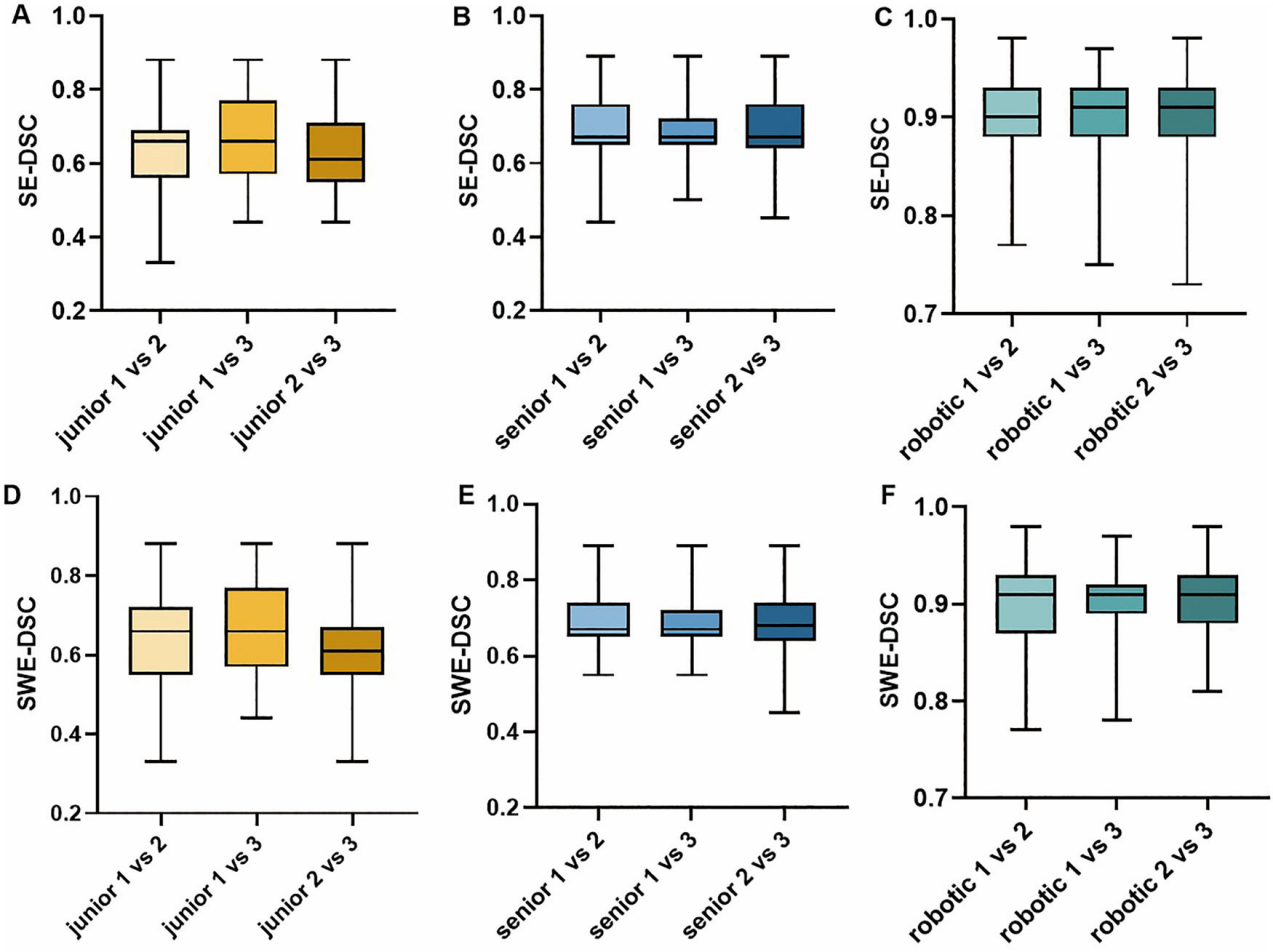
Intra-group spatial consistency of strain elastography (SE) and shear wave elastography (SWE) images assessed by Dice similarity coefficient (DSC) across three repeated acquisitions. **A**–**C** SE-DSC values for junior (**A**), senior (**B**), and robotic arm (**C**) groups. **D**–**F** SWE-DSC values for junior (**D**), senior (**E**), and robotic arm (**F**) groups. Box plots show DSC distributions for acquisition pairs (1 vs 2, 1 vs 3, and 2 vs 3) within each group. The robotic arm group demonstrated the highest spatial consistency in both modalities

In SE images (Fig. 5A–C), the DSC values among junior physicians were: 0.64 ± 0.12 (1 vs 2), 0.65 ± 0.15 (1 vs 3), and 0.62 ± 0.11 (2 vs 3); for senior physicians, the DSCs were 0.69 ± 0.09, 0.69 ± 0.08, and 0.68 ± 0.09, respectively. The robotic arm group showed significantly higher consistency: 0.91 ± 0.04, 0.89 ± 0.05, and 0.91 ± 0.04. Group comparisons showed that the robotic arm achieved a mean DSC of 0.90 ± 0.05, which was significantly higher than both the senior (0.69 ± 0.09) and junior groups (0.64 ± 0.13; all *p* < 0.001). The senior group also outperformed the junior group (*p* < 0.001).

In SWE images (Fig. 5D–F), the DSCs for the junior group were 0.63 ± 0.12, 0.66 ± 0.12, and 0.61 ± 0.11; for the senior group, values were 0.70 ± 0.09, 0.68 ± 0.08, and 0.68 ± 0.09. The force-controlled robotic arm group again demonstrated the highest consistency (0.90 ± 0.05, 0.89 ± 0.05, and 0.90 ± 0.04), with a mean DSC significantly greater than that of the senior (0.69 ± 0.09) and junior groups (0.64 ± 0.12; all *p* < 0.001). The senior group also showed better agreement than the junior group (*p* < 0.01).

### Intra-group consistency of Emax measurements in SWE images across radiologist groups

SWE and SE-based elasticity analyses demonstrated superior performance of the robotic arm, particularly in high-risk thyroid nodules. In the C-TIRADS 5 group, the robotic arm yielded the highest Emax values (89.83 ± 7.25 kPa), significantly outperforming manual radiologists and aiding in accurate identification of malignant nodules (*p* < 0.05; Table 2). For confirmed malignant nodules, Emax and SR values obtained by the robotic arm (Emax: 78.42 ± 7.56 kPa; SR: 1.21 ± 0.17) were significantly higher than those from both junior and senior physicians (*p* < 0.05; Table 3), indicating better differentiation capability. The force-controlled robotic arm demonstrated the highest consistency in both SR and Emax modes, with ICCs of 0.93 and 0.94 (95% CI: 0.85–0.96 and 0.88–0.97, respectively; both *p* < 0.01), significantly outperforming the senior physicians (0.84 and 0.81) and junior physicians (0.69 and 0.67, Table 4).

**Table 2:** SR and SWE values of thyroid nodules with different C-TIRADS categories assessed by different operators

	Junior		Senior		Robotic arm	
Categories	SR	SWE (kPa)	SR	SWE (kPa)	SR	SWE (kPa)
C-TIRADS 3 (*n* = 30)	0.94 ± 0.94	44.57 ± 10.64	0.98 ± 0.56	40.56 ± 8.62	0.99 ± 0.26	38.59 ± 2.92
C-TIRADS 4A/B/C (*n* = 55)	1.03 ± 0.83	54.58 ± 15.65	1.14 ± 0.45	66.84 ± 11.66	1.28 ± 0.42	75.83 ± 5.62
C-TIRADS 5 (*n* = 46)	1.06 ± 0.98	78.55 ± 16.36	1.18 ± 1.03	86.93 ± 14.2	1.31 ± 0.64	89.83 ± 7.25

*SR* strain ratio, *SWE* shear wave elastography

**Table 3:** Diagnostic measurements (SR and SWE) of benign and malignant thyroid nodules by different operators

	Benign (*n* = 33)		Malignant (*n* = 98)	
Operators	SR	SWE (kPa)	SR	SWE (kPa)
Junior	0.96 ± 0.94	43.57 ± 11.74	1.12 ± 0.74	67.84 ± 15.61
Senior	0.91 ± 0.34	45.57 ± 9.24	1.14 ± 0.51	70.42 ± 12.32
Robotic arm	0.89 ± 0.14	42.57 ± 5.61	1.21 ± 0.17	78.42 ± 7.56

*SR* strain ratio, *SWE* shear wave elastography

**Table 4:** Intraclass correlation coefficients (ICC) of Emax in SWE images among the three operator groups

Operator	ICC		95% CI		*P1*	*P2*
	SR	SWE	SR	SWE		
Junior	0.69	0.67	0.58–0.72	0.57–0.71	< 0.01	< 0.01
Senior	0.84	0.81	0.70–0.89	0.68–0.87	< 0.01	< 0.01
Robotic arm	0.93	0.94	0.85–0.96	0.88–0.97	< 0.01	< 0.01

### Image quality and acquisition efficiency

**Fig. 6:**
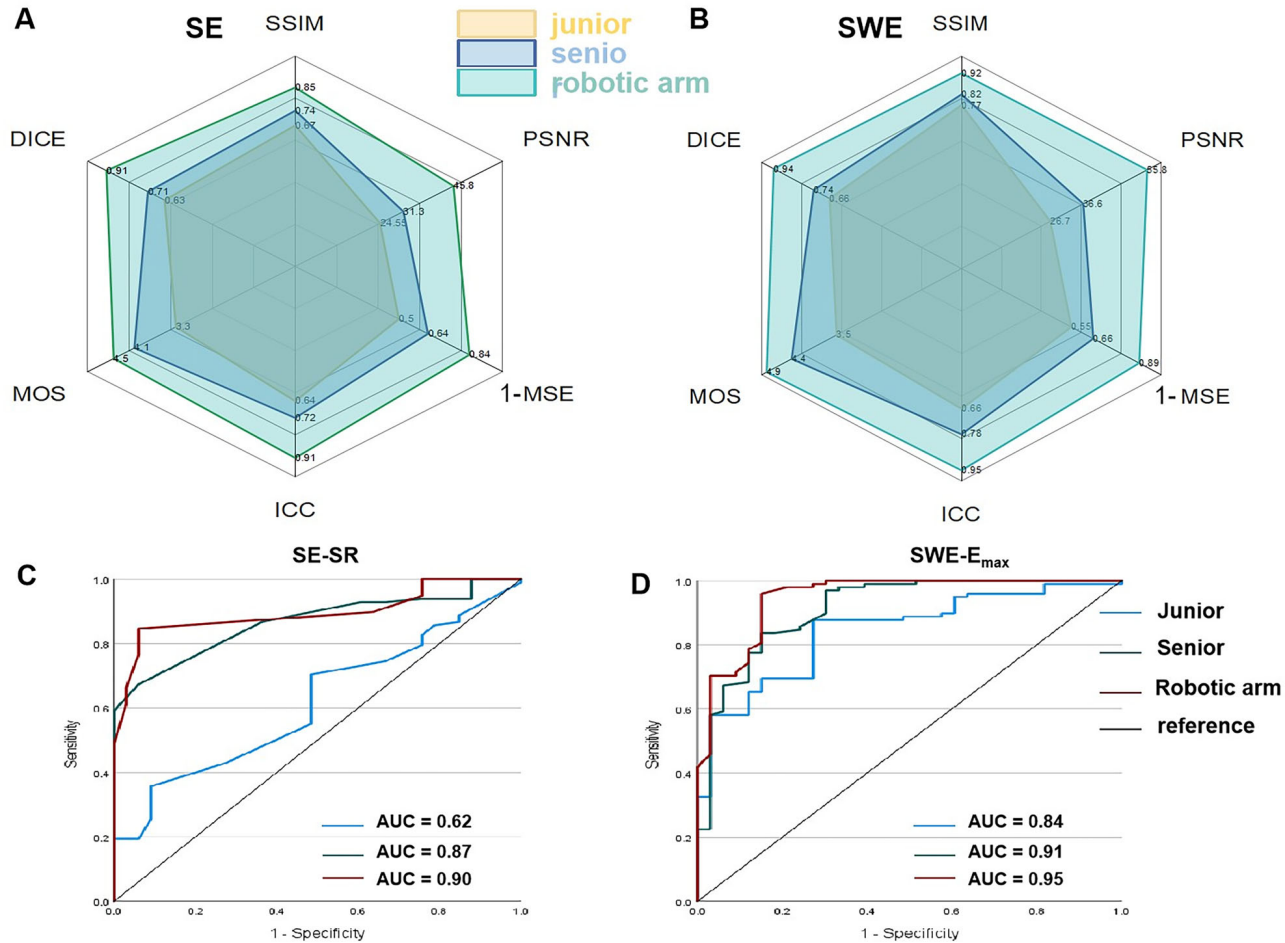
Comparison of three operator groups—junior physician, senior physician, and robotic arm—across image quality, consistency, and diagnostic performance metrics. **A**, **B** Radar plots of six normalized image quality and consistency metrics (SSIM, PSNR, MSE, MOS, DSC, ICC) in SWE (**A**) and SE (**B**) imaging show the robotic arm consistently outperformed both human operators; **C**, **D** ROC curves for differentiating benign and malignant thyroid nodules. In SE (**C**), AUCs were 0.62 (junior), 0.87 (senior), and 0.90 (robotic arm); in SWE using Emax (**D**), AUCs were 0.84 (junior), 0.91 (senior), and 0.95 (robotic arm). Significant differences were observed between groups (DeLong test, *p* < 0.05)

In both SE and SWE modes, the force-controlled robotic arm outperformed both junior and senior physicians across all image quality and consistency metrics. For SE images (Fig. 6A), the robotic arm group achieved the highest scores: SSIM = 0.94, PSNR = 45.8 dB, 1-MSE = 0.84, MOS = 4.9, DSC = 0.91, and ICC = 0.91. These values were significantly superior to those of the senior group (e.g., DSC = 0.71, ICC = 0.72) and junior group (DSC = 0.63, ICC = 0.64), with all differences reaching statistical significance (*p* < 0.001). Pairwise comparisons showed that the robotic arm group significantly outperformed the senior group (*p* < 0.01) and the junior group (*p* < 0.001), while senior physicians also showed improved performance over juniors (*p* < 0.05). Similarly, in SWE images (Fig. 6B), the robotic arm achieved SSIM = 0.92, PSNR = 55.8 dB, 1-MSE = 0.89, MOS = 4.9, DSC = 0.94, and ICC = 0.95-consistently higher than both physician groups. The junior group had the lowest performance in all metrics. All between-group differences were statistically significant (*p* < 0.001). Senior and robotic acquisitions were faster than those of the junior operator (P1 and P2 < 0.05), with no significant difference between senior and robotic groups (P3 = 0.68; Table S2).

### Diagnostic performance of manual and robotic arm elastography

ROC analysis demonstrated that the diagnostic performance of ultrasound elastography varied significantly across radiologist types (Fig. 6C, D). In the SE mode, the AUCs for the SR were 0.62 (95% CI, 0.51–0.72) for the junior physician group, 0.87 (95% CI, 0.81–0.93) for the senior physician group, and 0.90 (95% CI, 0.84–0.95) for the robotic arm group. The AUCs of the senior and robotic arm groups were significantly higher than those of the junior group (DeLong test, *p* < 0.05), with no significant difference observed between the senior physician and robotic arm groups (*p* > 0.05). In the SWE mode, the AUCs for the Emax were 0.84 (95% CI, 0.77–0.92) for the junior group, 0.91 (95% CI, 0.85–0.97) for the senior group, and 0.95 (95% CI, 0.90–0.99) for the robotic arm group. Pairwise comparisons revealed statistically significant differences between all groups (DeLong test, all *p* < 0.05).

## Discussion

This study comprehensively evaluated the performance of a force-controlled robotic arm in acquiring SE and SWE images for thyroid nodules, focusing on image quality, consistency, and diagnostic accuracy. Our findings demonstrated that the robotic system significantly outperformed both junior and senior physicians in producing high-quality, consistent, and diagnostically reliable elastography images.

In SWE imaging, the robotic arm group achieved superior objective metrics (SSIM = 0.91, PSNR = 41.63 dB, MSE = 3.06), and similar results were observed in SE (SSIM = 0.94, PSNR = 42.25 dB, MSE = 3.08). Subjective MOS scores were also the highest (SE: 4.1 ± 0.3; SWE: 4.3 ± 0.2), with all differences being statistically significant (all *p* < 0.001). These results suggest that the robotic system, through precise real-time adjustment of probe contact force, effectively mitigates radiologist-related variation and image noise, enhancing boundary definition and tissue contrast. This advantage was particularly pronounced in SE imaging, which is highly sensitive to pressure fluctuations. Consistent with previous studies highlighting the importance of standardized force application in elastography, our study further validates that robotic control can minimize image distortion and variability [16, 17].

In terms of intra-radiologist repeatability, the robotic arm achieved the highest DSC and ICC for Emax measurements (ICC = 0.94), significantly outperforming both physician groups (ICC = 0.81 and 0.67, respectively; *p* < 0.01). These results confirm that robotic acquisition offers superior reproducibility, particularly important for longitudinal assessments and dynamic monitoring scenarios. While earlier robotic ultrasound studies have reported improved image stability, our study is among the first to systematically quantify both image consistency and elasticity parameter reliability in SE and SWE modalities using a unified robotic system.

Radar plot analysis further reinforced the comprehensive advantage of the robotic system across six key performance metrics (SSIM, PSNR, MSE, MOS, DSC, ICC) in both SE and SWE modes. The robotic arm not only produced the highest normalized scores in all dimensions but also significantly reduced inter-radiologist variability, supporting its potential for standardized and optimized image acquisition. Beyond image quality, acquisition was efficient: the robotic system performed in line with the senior radiologist (*p* = 0.68) and faster than the junior operator (*p* < 0.01), underscoring that force control does not slow scanning.

In diagnostic evaluation, the robotic group achieved the highest AUCs for differentiating benign and malignant nodules (SWE: 0.95 vs 0.91 vs 0.84; SE: 0.90 vs 0.87 vs 0.62), with statistically significant differences even when compared to the senior physician. These results underscore the diagnostic value of standardized, force-controlled acquisition, especially in pressure-sensitive modalities such as SE.

In previous studies, telesonography robotic systems were primarily limited to conventional thyroid scanning. In this study, we enhanced the functionality of a telesonography platform by integrating a PID-based force control system, enabling its application to both SE and SWE. This advancement significantly improved image consistency and diagnostic performance, particularly in high-risk nodules and advanced C-TIRADS categories, highlighting the system’s potential for standardized and reproducible elastography in clinical practice.

Although the results are encouraging, several limitations should be acknowledged. First, the acquired elastography images still require manual interpretation by radiologists. Incorporating artificial intelligence for automated image analysis may further improve diagnostic accuracy in differentiating benign from malignant nodules and represents a promising direction for future research. Second, this single-center study involved an enriched cohort with a higher proportion of C-TIRADS 3–5/malignant nodules, focusing on technical feasibility for standardized acquisition and within-cohort discrimination. Next, we will conduct a prospective multicenter study with a larger sample and a higher proportion of benign nodules to validate performance in real-world populations and enhance generalizability. Despite these limitations, the robotic elastography system demonstrated stable and reproducible performance, underscoring its potential clinical value. Finally, optimizing AI diagnostic algorithms based on standardized imaging remains an important focus for future development.

## Conclusion

The force-controlled robotic arm demonstrated clear advantages in standardizing image acquisition, improving the consistency of elasticity measurements, and demonstrating higher diagnostic performance in thyroid nodule evaluation. Future integration with AI-driven navigation and interpretation may enable a transition from assistive robotic tools to intelligent diagnostic systems in ultrasound elastography.

## Supplementary information

ELECTRONIC SUPPLEMENTARY MATERIAL

Video 1

Video 2

Video 3

Video 4

## Data Availability

Research data regarding studies included and analysis are available on request.

## Abbreviations

AUC: Area under the curve
DSC: Dice similarity coefficient
ICC: Intraclass correlation coefficient
MOS: Mean opinion score
MSE: Mean squared error
PSNR: Peak signal-to-noise ratio
ROC: Receiver operating characteristic
SE: Strain elastography
SR: Strain ratio
SSIM: Structural similarity index
SWE: Shear wave elastography

## References

[1] Wang C, Gao Q, Zhang D et al (2024) Robotic assistance for standardized compression ultrasound elastography. In: Proceedings of the 2024 IEEE international conference on real-time computing and robotics (RCAR). IEEE, Alesund, pp 19–24

[2] Gilbertson MW, Anthony BW (2015) Force and position control system for freehand ultrasound. IEEE Trans Robot 31:835–849. 10.1109/TRO.2015.2429051

[3] Su K, Liu J, Ren X et al (2024) A fully autonomous robotic ultrasound system for thyroid scanning. Nat Commun 15:4004. 10.1038/s41467-024-48421-y 38734697 10.1038/s41467-024-48421-y PMC11519952

[4] Mena G, Montalvo A, Ubidia M et al (2023) Elastography of the thyroid nodule, cut-off points between benign and malignant lesions for strain, 2D shear wave real time and point shear wave: a correlation with pathology, ACR TIRADS and Alpha Score. Front Endocrinol 14:1182557. 10.3389/fendo.2023.1182557 10.3389/fendo.2023.1182557 PMC10313103 37396172

[5] Boers T, Braak SJ, Rikken NET et al (2023) Ultrasound imaging in thyroid nodule diagnosis, therapy, and follow-up: current status and future trends. J Clin Ultrasound 51:1087–1100. 10.1002/jcu.23430 36655705 10.1002/jcu.23430

[6] Swan KZ, Nielsen VE, Bonnema SJ (2021) Evaluation of thyroid nodules by shear wave elastography: a review of current knowledge. J Endocrinol Invest 44:2043–2056. 10.1007/s40618-021-01570-z 33864241 10.1007/s40618-021-01570-z

[7] Antico M, Sasazawa F, Wu L et al (2019) Ultrasound guidance in minimally invasive robotic procedures. Med Image Anal 54:149–167. 10.1016/j.media.2019.01.002 30928829 10.1016/j.media.2019.01.002

[8] Napoli ME, Freitas C, Goswami S et al (2018) Hybrid force/velocity control with compliance estimation via strain elastography for robot assisted ultrasound screening. In: Proceedings of the 2018 7th IEEE international conference on biomedical robotics and biomechatronics (Biorob). IEEE, Enschede, pp 1266–1273

[9] Yang C, Jiang M, Chen M et al (2021) Automatic 3-D imaging and measurement of human spines with a robotic ultrasound system. IEEE Trans Instrum Meas 70:1–13. 10.1109/TIM.2021.308511033776080

[10] Adams SJ, Burbridge BE, Badea A et al (2017) Initial experience using a telerobotic ultrasound system for adult abdominal sonography. Can Assoc Radiol J 68:308–314. 10.1016/j.carj.2016.08.002 28159435 10.1016/j.carj.2016.08.002

[11] Billings S, Deshmukh N, Kang HJ et al (2012) System for robot-assisted real-time laparoscopic ultrasound elastography. In: Holmes III DR, Wong KH (eds) Proceedings of SPIE medical imaging 2012: image-guided procedures, robotic interventions, and modeling, San Diego

[12] Cervi E, Bissacco D (2024) 5G-based robot-assisted remote ultrasound in vascular disease: a new era is coming! Int Angiol. 10.23736/S0392-9590.23.05138-6 10.23736/S0392-9590.23.05138-6 38078713

[13] Ipsen S, Wulff D, Kuhlemann I et al (2021) Towards automated ultrasound imaging—robotic image acquisition in liver and prostate for long-term motion monitoring. Phys Med Biol 66:094002. 10.1088/1361-6560/abf277 10.1088/1361-6560/abf277 33770768

[14] Czernuszewicz TJ, Aji AM, Moore CJ et al (2022) Development of a robotic shear wave elastography system for noninvasive staging of liver disease in murine models. Hepatol Commun 6:1827–1839. 10.1002/hep4.1912 35202510 10.1002/hep4.1912 PMC9234684

[15] Priester A, Natarajan S, Culjat M (2013) Robotic ultrasound systems in medicine. IEEE Trans Ultrason Ferroelect Freq Contr 60:507–523. 10.1109/TUFFC.2013.2593 10.1109/TUFFC.2013.2593 23475917

[16] Jiang Z, Salcudean SE, Navab N (2023) Robotic ultrasound imaging: state-of-the-art and future perspectives. Med Image Anal 89:102878. 10.1016/j.media.2023.102878 37541100 10.1016/j.media.2023.102878

[17] Jiang Z, Grimm M, Zhou M et al (2020) Automatic normal positioning of robotic ultrasound probe based only on confidence map optimization and force measurement. IEEE Robot Autom Lett 5:1342–1349. 10.1109/LRA.2020.2967682

