# Supplementary material for: Force-controlled robotic ultrasound elastography enhances diagnostic consistency for thyroid nodules
Source: Insights Imaging. 2026 Apr 16;17:101. doi: 10.1186/s13244-025-02181-5 (PMC13087058; doi:10.1186/s13244-025-02181-5)
Supplement: Supplementary file 1 — ELECTRONIC SUPPLEMENTARY MATERIAL [file 13244_2025_2181_MOESM1_ESM.pdf]

Force-Controlled Robotic Ultrasound Elastography Enhances Diagnostic Consistency for Thyroid Nodules

ELECTRONIC SUPPLEMENTARY MATERIAL

Insights Imaging (2025) Yan T, Xu L, Xie L, et al.

Appendix E1. Operator-specific workflows and operation times

All participants were examined in the supine position with the neck mildly extended. Target thyroid nodules were localized using conventional gray-scale ultrasound. Apply sufficient coupling agent between the probe and skin, then initiate SWE in zero-load condition (where the probe contacts only the coupling agent without visible soft tissue deformation). To minimize and standardize pre-compression bias across different acquisition techniques, each examination started from a zero-load alignment, and before switching to another technique, the probe was reset to the same no-preload contact and re-aligned accordingly. During robot-assisted acquisition, a closed-loop force control system was activated to maintain the contact force within a predefined range. For manual acquisitions, both senior and junior radiologists strictly followed a minimal-intervention protocol, ensuring light probe contact, avoiding near-field tissue thinning or deformation, and capturing frames only when the system's stability indicator confirmed optimal image quality. In robotic elastic imaging, the operator manually guides the robotic arm's end effector to move the probe holder along the skin surface to the target nodule's surface projection under zero-load alignment conditions. After engaging the closed-loop force control, SWE mode was activated and the image was saved once a stable elastogram was achieved. During manual elastography, senior and junior radiologists adhered to a standardized SWE protocol, maintaining minimal intervention while ensuring continuous image stability prior to image capture.

Acquisition time was recorded for all operator groups. Specifically, robotic-T, senior-T, and junior-T were defined as the time elapsed from “SWE activation” to acquisition of the “first stable elastographic image.”Image stability was assessed using the system's elasticity confidence indicator and verified by the same radiologist based on visual interpretability, defined as continuous and coherent color distribution without flickering or discontinuity. The image was saved once stability was achieved within this window. All three acquisition

modes—robotic, senior, and junior—were performed consecutively within the same examination session, with each subsequent mode initiated within one minute of the previous one to minimize patient movement and maintain procedural continuity. If stability criteria were not met on the first attempt, the scan was repeated after re-alignment under identical conditions; the first stable image obtained was used for timing and analysis.

For each participant, robotic-T, senior-T, and junior-T values were recorded, and process quality control was implemented throughout. All operators underwent standardized pre-study training and consistency assessment before patient enrollment to ensure procedural uniformity and measurement reliability.

Appendix E2. Objective and Reproducibility Metrics Definitions

SSIM (Structural Similarity Index): Measures image similarity in terms of luminance, contrast, and structural information. Values range from 0 to 1, with higher values indicating greater similarity.

PSNR (Peak Signal-to-Noise Ratio): Reflects image distortion; higher dB values represent lower noise and higher fidelity.

MSE (Mean Squared Error): Calculates the average squared intensity difference between paired pixels in test and reference images. Lower values indicate higher similarity.

MOS (Mean Opinion Score): A 5-point subjective quality rating scale (1 = very poor, 5 = very good) based on clarity, contrast, and anatomical boundaries.

DSC (Dice Similarity Coefficient): Evaluates spatial overlap between two segmentations, ranging from 0 (no overlap) to 1 (perfect match).

ICC (Intraclass Correlation Coefficient): Assesses the reliability of repeated quantitative measurements (e.g., Emax), with values >0.75 generally indicating good reproducibility.

**Table S1. Subjective Visual MOS Scoring Criteria (5-point Likert Scale)**	
1) Image clarity: Evaluate the edge sharpness and contrast of an image	1 to 5 points
2) Uniformity of elastic modulus distribution: Evaluate whether the color mapping of SE and SWE is uniform.	1 to 5 points
3) Degree of artifact interference: Analyze whether there are artifacts or unstable regions in SE and SWE images.	1 to 5 points
4) The image reflects the biomechanical characteristics of the nodule: The accuracy of the image reflecting the biomechanical characteristics of the lesion is subjectively judged by the assessor.	1 to 5 points

Table S2. Operator-specific SWE Procedure Durations

Operation	Operation times (s, median [IQR])	P1	P2	P3
Junior (junior-T)	15.1 [12.5, 18.1]	<0.01	<0.01	—
Senior (senior-T)	11.5 [9.2, 13.1]	<0.01	-	0.68
Robotic arm (robotic-T)	11.7 [7.1, 13.8]	—	<0.01	0.68

P1 = Junior vs Senior; P2 = Junior vs Robotic; P3 = Senior vs Robotic.
P values from pairwise post-hoc tests (Dunn–Bonferroni) following a Kruskal–Wallis overall test; "—" not applicable for the same comparison repeated across rows. Procedure duration was uniformly defined as the time from SWE activation to the first stable elastography image.

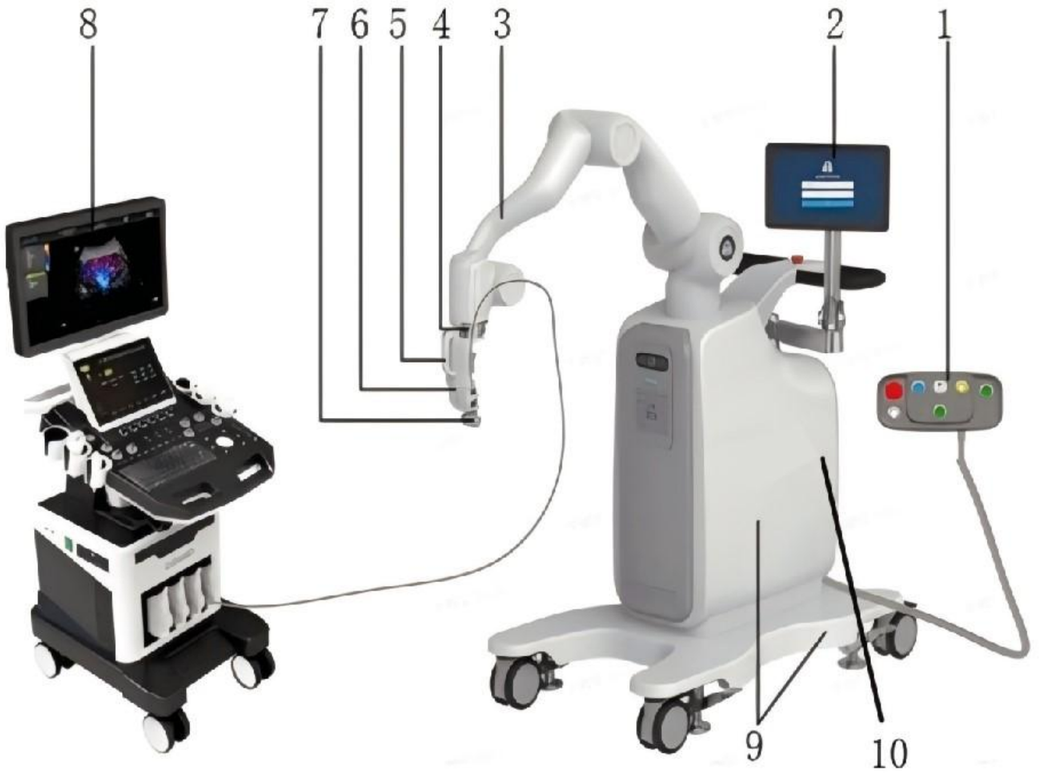

Figure S1. Diagram of the force-controlled robotic ultrasound system.
1: Handheld controller; 2: Touchscreen interface; 3: 7-DOF robotic arm; 4: 6-axis force/torque sensor; 5: Quick-release handle; 6: Probe gripper; 7: Ultrasound probe; 8: Ultrasound machine; 9: Mobile base; 10: Robot controller.

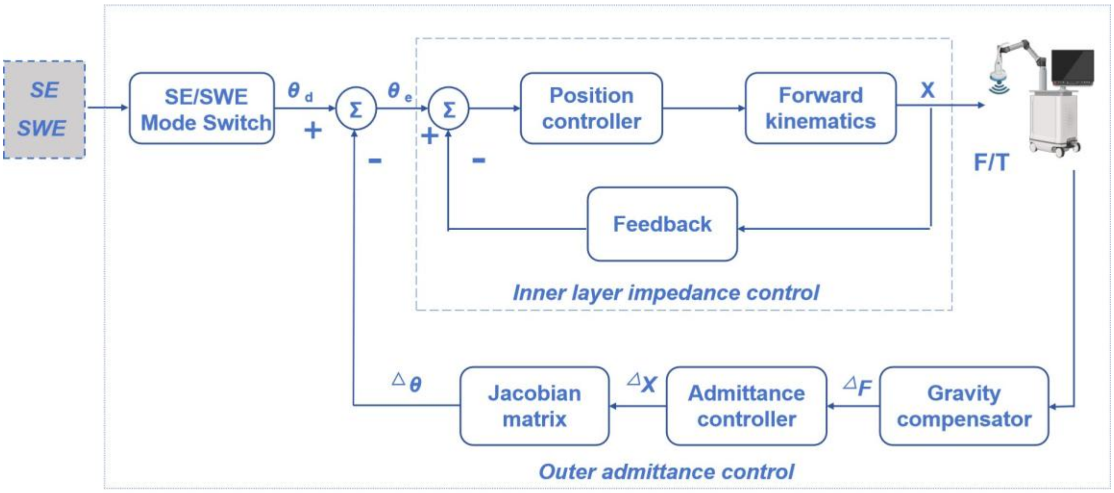

Figure S2. Control architecture of the force-controlled robotic arm for ultrasound elastography (SE/SWE) acquisition.

The system comprises an inner impedance control loop and an outer admittance control loop. The Mode Selector defines the desired joint angle (θ d) based on the selected imaging mode. The Position Controller regulates motion using the joint angle error (θ e =θ d− θ), where θ is the actual joint angle. Forward Kinematics converts joint positions into end-effector pose (X). The outer loop adjusts contact force in real time. Force/torque (F/T) feedback is used to compute force error (ΔF), from which the Admittance Controller generates a positional offset (ΔX). This offset is mapped to joint corrections (Δθ) via the Jacobian matrix. A Gravity Compensator offsets probe weight, ensuring stable and safe tissue contact during scanning.

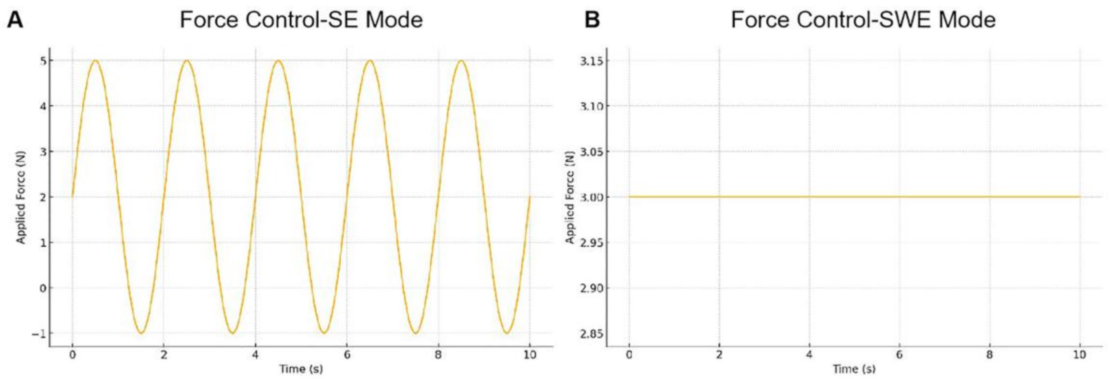

Figure S3. Force control strategies for robotic ultrasound elastography acquisition.
(A) SE mode (sinusoidal force control): the applied force varies sinusoidally over time with an amplitude of 3 N, frequency of 0.5 Hz, and a bias force of 2 N; (B) SWE mode (constant force control): the applied force remains constant at 3 N throughout the entire duration.

Vedio1 - SE - sinusoidal force
In SE mode, the Force-Controlled Robotic Arm applies sinusoidal wave force to obtain ultrasonic images.

Vedio2 - SWE - constant force
In the SWE mode, the Force-Controlled Robotic Arm applies a constant force to obtain ultrasonic images.

Vedio3 - SE
Dynamic ultrasound images obtained in the SE mode of the Force-Controlled Robotic Arm.

Vedio4 – SWE
Dynamic ultrasound images obtained in the SWE mode of the Force-Controlled Robotic Arm.
